# Study of Heavy Metal Levels among Farmers of Muda Agricultural Development Authority, Malaysia

**DOI:** 10.1155/2012/758349

**Published:** 2012-03-26

**Authors:** Ahmad Rohi Ghazali, Nur Ezzazulianie Abdul Razak, Mohd Sham Othman, Hidayatulfathi Othman, Ismarulyusda Ishak, Syarif Husin Lubis, Nihayah Mohammad, Zariyantey Abd Hamid, Zaliha Harun, Firdaus Kamarulzaman, Rozaini Abdullah

**Affiliations:** ^1^Biomedical Science Programme, Faculty of Health Sciences, School of Diagnostic and Applied Health Sciences, Universiti Kebangsaan Malaysia, Jalan Raja Muda Abdul Aziz, 50300 Kuala Lumpur, Malaysia; ^2^Environmental Health Programme, Faculty of Health Sciences, School of Diagnostic and Applied Health Sciences, Universiti Kebangsaan Malaysia, Jalan Raja Muda Abdul Aziz, 50300 Kuala Lumpur, Malaysia

## Abstract

Heavy metals, particularly cadmium, lead, and arsenic, constitute a significant potential threat to human health. This study was conducted to determine the levels of cadmium, lead, and arsenic in nail samples from farmers at Muda Agricultural Development Authority (MADA), Kedah, Malaysia, and evaluate factors that can contribute to their accumulations. A total of 116 farmers participated in this study. Inductively coupled plasma mass spectrometry (ICP-MS) was used to analyze concentration of heavy metals in the nail samples and questionnaires were given to participants to get demographic, health status, and their agricultural activities data. In this paper, the level of heavy metals was within the normal range and varies according to demographic factors. We found that there were significant correlations between working period with level of lead and arsenic (*r* = 0.315 and *r* = 0.242, resp., *P* < 0.01) and age with lead level (*r* = 0.175, *P* < 0.05). Our findings suggested that agricultural activities could contribute to the accumulation of heavy metals in farmers. Hence, the control of environmental levels of and human exposure to these metals to prevent adverse health effects is still an important public health issue.

## 1. Introduction

Farmers are exposed to a variety of pollutants particularly heavy metals that are released into the environment as a consequence of agricultural activities such as the use of pesticides and fertilizers. Some metals are extremely toxic to humans and the toxic heavy metals of greatest concern include cadmium, lead, and arsenic [1]. These heavy metals are incorporated into the organism via different routes and can then be stored and distributed in different tissues which lead to an internal bioconcentration that can induce different alterations, adverse effects, and/or diseases [2, 3]. 

 Therefore, it is important to determine the heavy metals concentration in occupationally exposed workers to monitor and assess their impact on human health. The use of human nail as a biomarker in occupational exposure to pollutants is an alternative biomarker besides blood and urine [4]. Nail is a good biomarker for several toxic elements in which the subjects had been exposed to these elements for the duration of 2 to 18 months [5]. Apart from that it is also a useful tool to measure the level of pollutants for long-term exposure [6]. According to International Atomic Energy Agency [7], human exposure to heavy metal at low level is a condition where it could cause poisoning and diseases, whereas accidental exposure at high level could cause serious effect immediately [8].

 In this study, the levels of cadmium, lead, and arsenic in nail samples from farmers were determined and those levels were then correlated with their demographic factors, blood pressure, and also their smoking working period and smoking habit.

## 2. Materials and Methods

### 2.1. Study Group

Subjects for this study were farmers working at the Wilayah III (Pendang, Kedah,) and Wilayah IV (Kota Sarang Semut, Kedah,) of MADA, Malaysia, who were chosen via universal sampling from a list of registered farmer under MADA authority. A total of 116 male Malay farmers took part in this study. Inclusion criteria of subjects for this study consisted of farmers that had been working more than one year and less than 60 years of age. The study protocol was approved and conducted in accordance with the Ethical Principles for Medical Research Involving Human Subjects as defined by the research institution.

### 2.2. Questionnaire

Questionnaires were used to collect demographic data and factors that can influence the levels of heavy metals in the samples. Each subject was asked on the sections listed in the questionnaire which included (A) personal background, (B) awareness of illness, and (C) agricultural activities.

### 2.3. Sample Analysis

All glassware and plastic equipments were immersed in nitric acid solution overnight to avoid contamination. Then, all the equipments were rinsed with deionized water, dried, and stored appropriately. Soil and dirt were removed from the nail sample using the method recommended by the International Atomic Energy Agency [9] with slight modifications [10, 11]. The samples were then rinsed thoroughly with deionized water and placed in the desiccator for drying processes and stored in sealed plastic bags at room temperature until further processing was carried out.

 Then, a total of 10–20 mg samples were weighed using electronic scales and placed in a porcelain bowl that had been cleaned. Samples were then heated on a heating plate (Stuart Scientific, UK) until the ash was formed [11]. After cooling, the ash samples were moistened with 0.5 mL deionized water, and then 1 mL of nitric acid and 0.5 mL perchloric acid were added accordingly. Then, the samples were heated up to dry on a heating plate (Stuart Scientific, UK) at a temperature of 20°C. About 0.5 mL of nitric acid and deionized water are added into the resulting residues and heated for 5 minutes until a clear solution was formed. This solution was rinsed in 10 mL volumetric flask with deionized water until the final volume of 10 mL. Samples were stored in metal-free plastic tubes at room temperature until the determination of the level of heavy metals was conducted using ICP-MS (PerkinElmer, USA) [12].

### 2.4. Statistical Analysis

Descriptive analysis was conducted to obtain means, standard deviations, and range of heavy metals in the nail samples. We employed one-way ANOVA test to compare the means between heavy metals according to blood pressure and smoking habit. On the other hands, to find the correlation of the heavy metals with age and working period, we employed the Spearman correlation test because the data for these variables were not normally distributed. All the data collected were analyzed statistically using SPSS software version 17.0.

## 3. Results and Discussion

The influence of environmental pollution on human health can be determined in terms of biomonitoring of the metabolically inactive tissues such as nails due to easy of sample collection, transportation, storage, and preparation for analysis. Blood and urine could be alternative biomarkers but these samples are meant for short-term exposure [4].

In our findings, the average levels of cadmium, lead, and arsenic (*μ*g/g) in the nail samples from the MADA farmers are presented in Table 1. Since there was an insufficient reference on baseline data of the levels of heavy metals in Malaysian population study, we compared our results with other similar studies from other countries.  Table 2 shows comparison of means ± standard deviations of cadmium, lead, and arsenic (*μ*g/g) levels in nail samples from references with similar studies from other countries.

Cadmium level in this study was found higher than the study conducted by Samanta et al. [5], but lower than the level of cadmium in other studies. Study by Samanta et al. [5] showed that the subjects were exposed to heavy metals through rice consumption from paddy fields that could be polluted by heavy metals from a nearby mining area. For the concentration of lead, our result was higher than the study conducted by Mortada et al. [14]. For the level of arsenic, our study showed higher levels compared to the previous studies showed in Table 2. The levels of heavy metals in our study could be due to the use of pesticides and fertilizers in the agricultural activities [13, 15].

Table 3 shows the average levels of cadmium, lead, and arsenic in farmers according to their blood pressure and smoking habits. Results from this study showed that there was no significant difference in the levels of heavy metals in normal and high blood pressure groups. A study conducted by Sukumar and Subramanian [16] also found that there was no significant difference for cadmium and lead levels among subjects with high blood pressure and normal blood pressure. For arsenic, there was also no significant difference between subjects with high blood pressure and normal blood pressure groups. Other factors such as dietary intake might influence the levels of heavy metals in the nail samples of the subjects in our study.

Smoking is also associated with high blood pressure and heavy metal content [17]. This could be due to the cadmium and other heavy metals content in the cigarettes [18]. Results from Sukumar and Subramanian [16] and Mortada et al. [14] studies found that cadmium levels were significantly higher among subjects who smoked. However, this study showed that levels of cadmium did not differ significantly (*P* > 0.05) among smokers and nonsmokers groups. Levels of lead and arsenic also showed no significant difference (*P* > 0.05) between smokers and nonsmokers. Studies conducted by Sukumar and Subramanian [16] also found that lead levels were not influenced by smoking.

Table 4 shows the relationships between the farmers' age and level of heavy metals from their nail samples. There was no significant relationship between both cadmium and arsenic levels with age (*r* = − 0.045 and *r* = 0.124, resp., *P* > 0.05) according to the Spearman correlation test. However, the analysis of the relationship between the age and levels of lead showed a significant relationship (*r* = 0.175, *P* < 0.05). A study conducted by Rodushkin and Axelsson [19] showed that age did not influence the levels of heavy metals in the nail samples. Lead levels were also found to be increased among older individuals than younger individuals [14].

Table 4 also shows the relationships between working period as farmers and level of heavy metals in their nail samples. This study found that there was no relationship between cadmium level and their working period as farmers (*r* = 0.0117, *P* > 0.05). Cadmium accumulates primarily in liver and kidney in which it would bind to metallothionein [20]. This might be the reason for the insignificant difference between the cadmium level in the nail samples and their working period. However, there was a significant relationship between working period as a farmer and the level of lead and arsenic among MADA farmers (*r* = 0.315 and *r* = 0.242, resp., *P* < 0.01). Faridah et al. [21] stated that cadmium and lead were potential bioaccumulators because of their long half-lives. Most farmers who had been working for 20–30 years and exposed to pesticides and fertilizers could increase the levels of heavy metals in their bodies [15].

In addition, data collected from questionnaires showed that almost 95% of subjects used protective equipment when working. Protective equipment is a tool to reduce exposure and prevent harm including heavy metals material from entering the body. Ironically, in this study we found that the level of arsenic was still high among the subjects. While assessing the heavy metals exposure, other factors such as nutrition, socioeconomic status, exposure conditions, genetic variability and susceptibility, have to be considered for a realistic approach.

## 4. Conclusion

There were significant correlations between heavy metals with subject's age and working period as farmers. However, there was no significant correlation between heavy metals and their blood pressure and smoking habits. Hence, exposure of heavy metals to the farmers could be due to the use of pesticides and fertilizers in various agricultural activities. The control of environmental levels of and human exposure to these metals to prevent adverse health effects is still an important public health issue and other alternatives to control use of fertilizers and reducing the pesticides applications have to be implemented.

## Figures and Tables

**Table 1:** Levels of cadmium, lead, and arsenic (*μ*g/g) in the nail sample of MADA farmers.

Metal	Mean concentration (mean ± SD)
Cadmium	0.874 ± 0.746
Lead	6.611 ± 5.170
Arsenic	7.801 ± 3.184

**Table 2:** Comparison of means ± standard deviations of cadmium, lead, and arsenic (*μ*g/g) levels in nail samples from references with similar studies from other countries.

References	Mean concentration (mean ± SD)		
	Cadmium (Cd)	Plumbum (Pb)	Arsenic (As)
Present study	0.874 ± 0.746	6.611 ± 5.170	7.801 ± 3.184
[13]	2.50 ± 1.70	9.2 ± 1.5	ND
[14]	1.35 ± 0.85	4.82 ± 2.61	ND
[5]	0.32 ± 0.09	10.99 ± 2.04	7.24 ± 1.28

ND : not detected.

**Table 3:** Comparison of average levels of cadmium, lead, and arsenic (mg/g) in farmers according to their blood pressure and smoking habits.

Parameter	Mean concentration (mean ± SD)		
	Cadmium (Cd)	Plumbum (Pb)	Arsenic (As)
*Blood pressure *			
Normal blood pressure	0.784 ± 0.687	6.222 ± 5.210	7.453 ± 3.047
High blood pressure	1.012 ± 0.818	6.999 ± 5.045	8.267 ± 3.371
*Smoking habit *			
Smokers	0.921 ± 0.787	6.693 ± 5.228	7.798 ± 3.025
Nonsmokers	0.756 ± 0.655	6.285 ± 5.680	7.634 ± 3.716

**Table 4:** Correlation between heavy metals with age and the working period of the farmers.

Heavy metal	Correlations, *r*	
	Age	Working period
Cadmium	−0.045	0.117
Lead	0.175*	0.315**
Arsenic	0.124	0.242**

There is significant correlation between heavy metals with both working period and age (*P < 0.05, **P < 0.01).

## References

[1] Zanini E, Bonifacio E, Cuttica GC (1992). Heavy metals in soils near a steel-making industry: a case study in a complex valley situation in Italy. *Journal of Environmental Science and Health A*.

[2] Chen Y, Wang C, Wang Z (2005). Residues and source identification of persistent organic pollutants in farmland soils irrigated by effluents from biological treatment plants. *Environment International*.

[3] Singh KP, Mohan D, Sinha S, Dalwani R (2004). Impact assessment of treated/untreated wastewater toxicants discharged by sewage treatment plants on health, agricultural, and environmental quality in the wastewater disposal area. *Chemosphere*.

[4] Wang T, Fu J, Wang Y, Liao C, Tao Y, Jiang G (2009). Use of scalp hair as indicator of human exposure to heavy metals in an electronic waste recycling area. *Environmental Pollution*.

[5] Samanta G, Sharma R, Roychowdhury T, Chakraborti D (2004). Arsenic and other elements in hair, nails, and skin-scales of arsenic victims in West Bengal, India. *Science of the Total Environment*.

[6] Batista BL, Rodrigues JL, Nunes JA, Tormen L, Curtius AJ, Barbosa F (2008). Simultaneous determination of Cd, Cu, Mn, Ni, Pb and Zn in nail samples by inductively coupled plasma mass spectrometry (ICP-MS) after tetramethylammonium hydroxide solubilization at room temperature: comparison with ETAAS. *Talanta*.

[7] IAEA Activation analysis of hair as an indicator of contamination of man by environmental trace element pollutant.

[8] Needham LL, Patterson DG, Barr DB, Grainger J, Calafat AM (2005). Uses of speciation techniques in biomonitoring for assessing human exposure to organic environmental chemicals. *Analytical and Bioanalytical Chemistry*.

[9] Ryabukhin YS (1978). Activation analysis of hair as indicator of contaminantion of man by environmental trace element pollutants.

[10] Saiki M (2001). Determination of trace elements in human nail clippings by neutron activation analysis. *Journal of Radioanalytical and Nuclear Chemistry*.

[11] Bergomi M, Vinceti M, Nacci G (2002). Environmental exposure to trace elements and risk of amyotrophic lateral sclerosis: a population-based case-control study. *Environmental Research*.

[12] Ming Y, Bing L (1998). Determination of rare earth elements in human hair and wheat flour reference materials by inductively coupled plasma mass spectrometry with dry ashing and microwave digestion. *Spectrochimica acta B*.

[13] FAO

[14] Mortada WI, Sobh MA, El-Defrawy MM, Farahat SE (2002). Reference intervals of cadmium, lead, and mercury in blood, urine, hair, and nails among residents in Mansoura city, Nile Delta, Egypt. *Environmental Research*.

[15] Wei B, Yang L (2010). A review of heavy metal contaminations in urban soils, urban road dusts and agricultural soils from China. *Microchemical Journal*.

[16] Sukumar A, Subramanian R (2007). Relative element levels in the paired samples of scalp hair and fingernails of patients from New Delhi. *Science of the Total Environment*.

[17] WHO (2003). *International Society of Hypertension (ISH) Statement on Management of Hypertension*.

[18] Jarup L, Akesson A (2009). New Insights into the mechanisms of cadmium toxicity-advances in cadmium research. *Toxicology and Applied Pharmacology*.

[19] Rodushkin I, Axelsson D (2000). Application of double focusing sector field inductively couple plasma-mass spectrometry multi-elemental characterization of human and nail part II. A study of inhibitants of northen Sweeden. *Science of The Total Environment*.

[20] Goering PL, Waalkes MP, Klaassen CD, Goyer RA, Cherian MG (1994). Handbook of experimental pharmacology. *Toxicology of Metals, Biochemical Effects*.

[21] Faridah HW, Wilson N, Murugi J, Wanjau R (2008). Use of human nails as bio-indicators of heavy metals environmental exposure among school age children in Kenya. *Science of the Total Environment*.

